# Keratin 76 Is Required for Tight Junction Function and Maintenance of the Skin Barrier

**DOI:** 10.1371/journal.pgen.1004706

**Published:** 2014-10-23

**Authors:** Tia DiTommaso, Denny L. Cottle, Helen B. Pearson, Holger Schlüter, Pritinder Kaur, Patrick O. Humbert, Ian M. Smyth

**Affiliations:** 1 Department of Biochemistry and Molecular Biology, Monash University, Melbourne, Australia; 2 Research Division, The Sir Peter MacCallum Cancer Centre, Melbourne, Australia; 3 The Sir Peter MacCallum Department of Oncology, University of Melbourne, Melbourne, Australia; 4 Department of Anatomy & Neuroscience, University of Melbourne, Melbourne, Australia; 5 Department of Biochemistry and Molecular Biology, University of Melbourne, Melbourne, Australia; 6 Department of Pathology, University of Melbourne, Melbourne, Australia; 7 Department of Anatomy and Developmental Biology, Monash University, Melbourne, Australia; Stanford University School of Medicine, United States of America

## Abstract

Keratins are cytoskeletal intermediate filament proteins that are increasingly being recognised for their diverse cellular functions. Here we report the consequences of germ line inactivation of *Keratin 76* (*Krt76*) in mice. Homozygous disruption of this epidermally expressed gene causes neonatal skin flaking, hyperpigmentation, inflammation, impaired wound healing, and death prior to 12 weeks of age. We show that this phenotype is associated with functionally defective tight junctions that are characterised by mislocalization of the integral protein CLDN1. We further demonstrate that KRT76 interacts with CLDN1 and propose that this interaction is necessary to correctly position CLDN1 in tight junctions. The mislocalization of CLDN1 has been associated in various dermopathies, including the inflammatory disease, psoriasis. These observations establish a previously unknown connection between the intermediate filament cytoskeleton network and tight junctions and showcase *Krt76* null mice as a possible model to study aberrant tight junction driven skin diseases.

## Author Summary

The generation of knockout mice is a central approach to studying gene function. We have examined the consequences of the germ line inactivation of *Keratin 76* in mice and in doing so we reveal a previously undescribed mechanism by which keratin intermediate filaments regulate cellular interactions and tissue homeostasis. Our study supports an emerging body of evidence which challenges the classical view of the keratin intermediate filaments as simple structural proteins, highlighting *Krt76* as a gene whose function is indispensable for barrier function and skin wound repair as a result of its novel interaction with tight junction complexes. This study identifies a previously unknown and critical link between intermediate filaments and tight junctions where intermediate filament dysfunction influences skin disease.

## Introduction

The epidermis provides a stable and selectively permeable barrier essential to terrestrial life. Together with microfilaments and microtubules, intermediate filaments (IFs) make up the major components of the epidermal cytoskeleton. Keratins are the largest subgroup of the IF proteins and comprise the major structural proteins in epithelial cells [1]. Keratins are composed of a central, filament forming, alpha-helical rod domain of ∼310 amino acids that is flanked by non-helical head and tail domains [1], [2], [3], [4], [5]. They act as a flexible scaffold enabling cells to resist physical stress. Consequently, defects in IFs can lead to cell fragility and are linked to a wide array of genodermatoses and cancers [5], [6]. The classical view that keratins simply provide a structural scaffold has been challenged by recent studies demonstrating their increasingly specialised and diverse functions [7]. These include protection from apoptosis [8], [9] and injury [10], regulation of epithelial polarity [11], [12] and influence on cell size and protein translation[10], [13], [14], [15].

The functional integration of cytoskeletal elements and cellular junctions is critical for the establishment and maintenance of the epidermal barrier. Tight junctions (TJ) form a seal between cells which make up the layers of the epidermis [16]. This barrier is selectively permeable, allowing passage of small molecules, but restricting water loss, and allowing for antigen sampling by immune cells [16], [17], [18]. TJs are composed of scaffolding and adhesion molecules including claudins, junctional adhesion molecules and occludins. Defective tight junction organization has been linked to compromised barrier function [17] and the development of various dermopathies including psoriasis [19], [20]. The TJs are thought to interact with the IF network by binding of a number of integral or associated TJ proteins that complex to to F-actin [21] but their associations, if any, with the keratin IF network are unclear.

In this report we have studied the effects of *Krt76* disruption in mice and demonstrate that the KRT76 protein is essential for postnatal survival beyond ∼3 months of age. Loss of KRT76 leads to the acquisition and infection of skin wounds which fail to properly resolve over time. This phenotype correlates with observations showing that the gene is up-regulated during normal wound healing and is required for this process. At a mechanistic level we show that loss of KRT76 is associated with defective tight junction function through the mislocalization of Claudin1 (CLDN1), an integral TJ component which we show binds to KRT76. These findings identify a critical new relationship between the IF network and TJs which we propose is essential for epidermal homeostasis.

## Results

### Loss of *Krt76* causes gross epidermal defects and results in lethality

**Figure 1:**
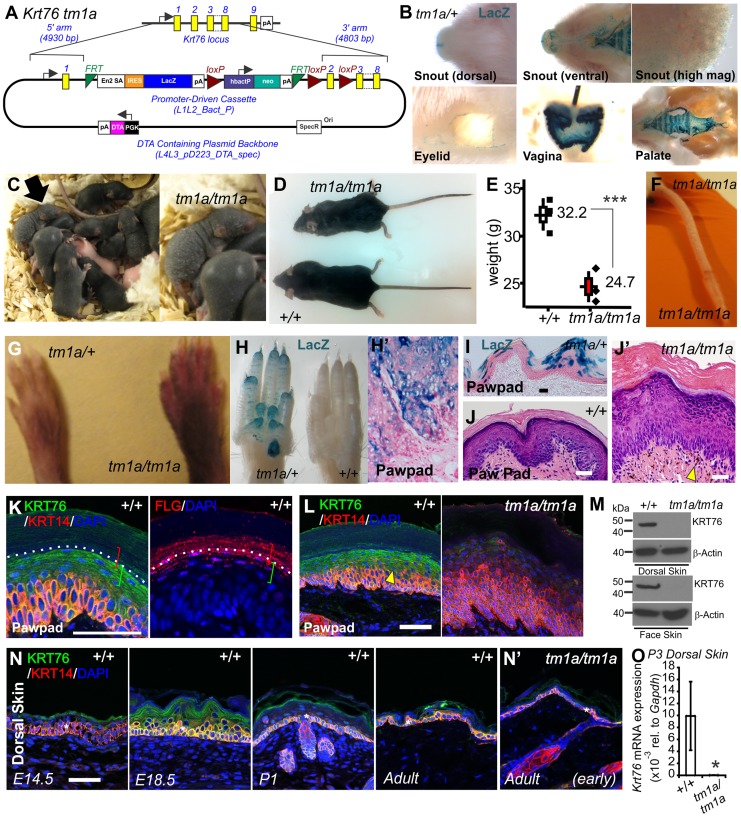
*Krt76* gene trap disruption causes gross epidermal defects. (A) Schematic showing *Krt76* gene trap (knock-out first) targeting construct. (B) Whole mount LacZ staining of *Krt76^tm1a/+^* reporter mice, shows *Krt76* expression in the dorsal and ventral snout and palate, eyelid, and vagina. (C) Mice homozygous for *Krt76* gene trap disruption (*Krt76^tm1a/tm1a^*) exhibit flaky skin following birth (see arrow-insert). Adult *Krt76^tm1a/tm1a^* mice exhibit a scruffy coat and smaller body weight (n = 3 males, age 9 weeks, ***p<0.004) (D, E), as well as tail scaling (F). *Krt76^tm1a/tm1a^* mice exhibit paw pad hyperpigmentation (G), concurring with regions of LacZ reporter expression (H). LacZ expression within paw pads is detected in exocrine glands (H′) and suprabasal epidermal layers (I). (J, J′) Haemotoxylin and Eosin (H&E) staining of paw pads from WT (J) and *Krt76^tm1a/tm1a^* (J′) mice. Yellow arrowheads indicate abnormal dermal pigmentation. (K, L) Immunofluorescence analysis with indicated antibodies in wild type and *Krt76^tm1a/tm1a^* mouse paw pad. Samples are counter stained with nuclear dye DAPI (4',6-diamidino-2-phenylindole). Coloured brackets indicate approximate distribution of FLG and KRT76 expression around the granular layer. (M) Western blot analysis of WT and *Krt76^tm1a/tm1a^* dorsal skin and face skin extracts. (N) Immunofluorescence analysis with anti-KRT76 and anti-K14 antibodies in wild type mouse dorsal skin at E14.5, E18.5, P1 and adult time points and adult *Krt76^tm1a/tm1a^* dorsal skin (N′). Asterisks indicate non-specific basal layer staining. (O) *Krt76* mRNA qRT-PCR analysis of p3 dorsal skin relative to *Gapdh*. Scale bars represent 50 µm.

As part of the Wellcome Trust Sanger Institute (WTSI) Mouse Genetics Programme [22], we screened the skin of the mutant mouse strains generated. This skin screen is discussed in an accompanying article in this issue of PLoS Genetics [23]. From this screen we identified significant cutaneous defects in mice homozygous for the gene trap “knockout first” [24] allele of *Keratin 76* (*Krt76^tm1a(KOMP)Wtsi^* hereafter *Krt76^tm1a^*) (Figure 1A) [23]. These animals have a splice acceptor-LacZ reporter integrated upstream of floxed exon 2 that allows gene expression to be traced whilst disrupting gene function. Quality control of this mutant allele and correct genome positioning has been confirmed by long range PCR (http://www.sanger.ac.uk/mouseportal/search?query=krt76). *Krt76* expression has previously been reported in the palatal and gingival epithelium [25]. By utilising the integrated LacZ reporter in our *Krt76^tm1a/+^* model we confirmed expression at these locations but also detected previously unreported expression in the vagina and the eyelid (Figure 1B).

*Krt76^tm1a^* mice were then further back-crossed onto a C57BL/6 genetic background and bred back to homozygosity to determine the full consequences of *Krt76* disruption. *Krt76^tm1a/tm1a^* neonates exhibit flaky skin (Figure 1C, arrow-insert), although these defects diminish somewhat with the emergence of hair follicles. After weaning, mutant mice are distinguished by their unkempt, dull coats and smaller body size (Figure 1D, E) and by scaling of skin of the tail (Figure 1F) [26]. *Krt76^tm1a/tm1a^* mice also show abnormal paw pad hyperpigmentation (Figure 1G) which corresponds with *Krt76* expression as reported by LacZ, which is observed throughout the stratified epidermal layers and in the exocrine glands (Figure 1H, H′, I).

Haematoxylin and Eosin (H&E) staining of wild type (WT) and *Krt76^tm1a/tm1a^* paw pads revealed an overall epidermal thickening, with reduced granular layer compaction and an increased cornified layer (Figure 1J, J′). Pigment was also observed in the dermis (Figure 1J′ see arrowhead). To further explore KRT76 expression, we performed immuno-staining on paw pad skin using antibodies raised against human KRT76 epitopes that are predicted to be disrupted in *tm1a* animals. Specific KRT76 expression was highest in the granular cell layer where it overlapped with Filaggrin (FLG) in serial sections (Figure 1K). This site of expression correlates with β-galactosidase activity detected via the integrated reporter gene (Figure 1I). Importantly granular layer staining was absent from *Krt76^tm1a/tm1a^* animals (Figure 1L) and Western blots from epidermal extracts of the mid-dorsum and face epidermis confirmed that the *tm1a* allele results in complete loss of KRT76 protein (Figure 1M). We did detect low levels of immuno-staining in basal keratinocytes in *Krt76^tm1a/tm1a^* skin which was slightly reduced compared to wild type mice when imaging was performed using the same confocal settings. Given the unequivocal Western results, one interpretation is that this basal signal is a combination of non-specific cross reactivity and low levels of *bona fide* expression at this location. However, we cannot exclude the possibility that this change instead relates to alterations in expression of the cross-reacting species that might occur as a consequence of loss of KRT76. Further studies, perhaps using different antibodies, would be required to confirm this.

To examine a developmental role for the gene we profiled protein expression during embryonic and postnatal skin development, showing increasing levels of protein associated with the differentiation of the skin during late embryonic development, followed by a subsequent reduction in expression levels after birth (Figure 1N). Importantly though, low levels of KRT76 were still detectable in the spinous and granular cell layers in intact adult dorsal skin (Figure 1N). As expected, KRT76 protein was absent in *Krt76 ^tm1a/tm1a^* animals by both immunofluorescence (compare Figure 1N with Figure 1N′) and qRT-PCR (Figure 1O), further validating this allele as a *bona fide* knockout model. This profile also suggests KRT76 may have a role in the later steps of keratinocyte differentiation, an observation which correlates with the flaky skin phenotypes observed in *Krt76^tm1a/tm1a^* neonates (Figure 1C).

### *Krt76* is associated with barrier maturation wound healing

**Figure 2:**
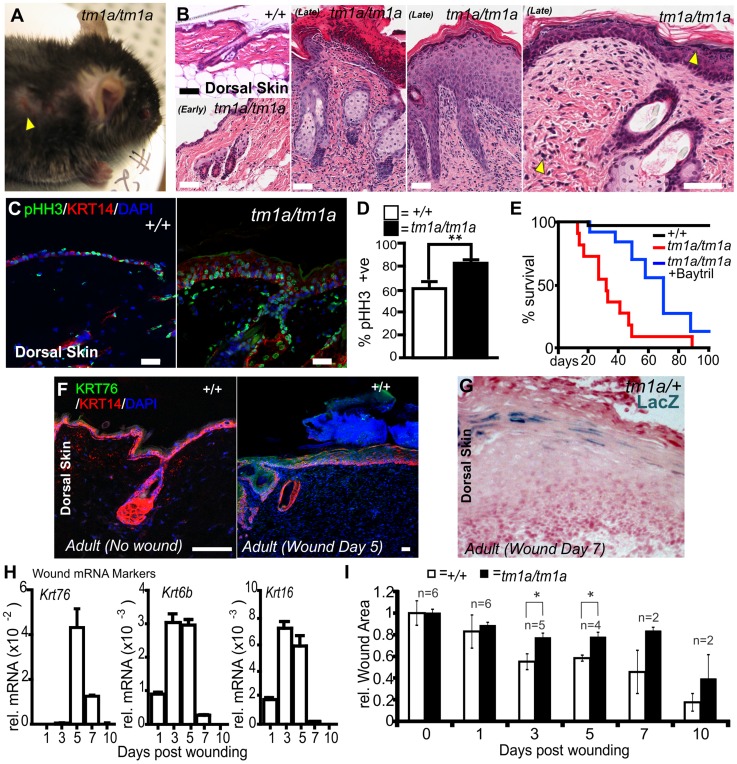
*Krt76* is required for normal wound healing. (A) *Krt76^tm1a/tm1a^* gene trap mice show spontaneous wounds around the eyes and shoulders (sites of grooming). (B) H&E staining of dorsal skin from adult WT, early and late phenotype *Krt76^tm1a/tm1a^* mice. Yellow arrowhead indicates abnormal pigmentation in the dermis and epidermis. (C) Immunofluorescence phospho-histone H3 (pHH3) analysis of wild type and *Krt76^tm1a/tm1a^* mouse dorsal skin shows (D) increased proliferation/pHH3 positive cells (*p = 0.005*). E) *Krt76^tm1a/tm1a^* mice die progressively from ∼2 weeks after birth with no animals surviving beyond 12 weeks of age. Treatment with Baytril reduces morbidity and mortality. (F) Immunofluorescence analysis with anti-KRT76 and anti-K14 antibodies in non-wounded and wounded wild type dorsal skin after 5 days. (G) LacZ staining of the wounded skin from *Krt76^tm1a/+^* reporter mice at 7 days post wounding. (H) *Krt76, Krt6b* and *Krt16* mRNA qRT-PCR analysis of wounded skin relative to *Gapdh*, over 10 days. (I) Quantification of wound closure in wild type and *Krt76^tm1a/tm1a^* mice over 10 days. *p<0.05, Error bars are S.E.M. Scale bars represent 50 µm.

As they age, *Krt76^tm1a/tm1a^* mice develop spontaneous wounds that fail to heal, especially on the dorsal skin around sites of active grooming (Figure 2A, see arrow head). Histological examination showed no obvious phenotypic change in young *Krt76^tm1a/tm1a^* mice prior to significant wound acquisition (which we refer to as the “early” phenotype), but large scabs, immune dermal infiltrates, extreme IFE thickening (Figure 2B) and hyperpigmentation in the dermis and epidermis develop over time (arrrowheads, Figure 2B). Phospho-histone H3 staining demonstrated a hyperproliferative response in these mice (Figure 2C, D). The morbidity associated with loss of KRT76 is such that animals rarely survive beyond 12 weeks of age. To assess whether cutaneous bacterial infection of these spontaneous wounds may exacerbate morbidity, we treated *Krt76^tm1a/tm1a^* mice with a broad spectrum antibiotic (Baytril) and observed a considerable improvement in lifespan (median survival  = 70 days versus 32 (p<0.04)) (Figure 2E).

The wounding phenotypes associated with *Krt76^tm1a/tm1a^* mice led us to examine whether KRT76 was directly involved in the healing of induced wounds. As a first step in addressing this question we sampled dorsal skin from *Krt76^tm1a/+^* and WT mice 1, 3, 5, 7, and 10 days after wounding by punch biopsy to examine gene expression. Immunofluorescence staining for KRT76 showed an up-regulation of KRT76 protein in the healing wound 5 days after injury (Figure 2F). This was confirmed by LacZ staining in *Krt76^tm1a/+^* wound sections (Figure 2G). Expression profiling by qRT-PCR in WT mice confirmed *Krt76* mRNA upregulation in response to wounding, with a profile slightly delayed in comparison to the “classical” wounding keratins *Krt6b* and *Krt16* (Figure 2H). Similar punch biopsy experiments in the dorsal epidermis in *Krt76^tm1a/tm1a^* mice resulted in a significant impairment in wound closure at day 3 and day 5, correlating with the peak of *Krt76* expression in the wound (Figure 2I). These observations indicate that that KRT76 is normally upregulated in response to skin damage and is required to facilitate wound healing during the latter phases of this process.

### Biochemical analysis of *Krt76* disrupted skin

**Figure 3:**
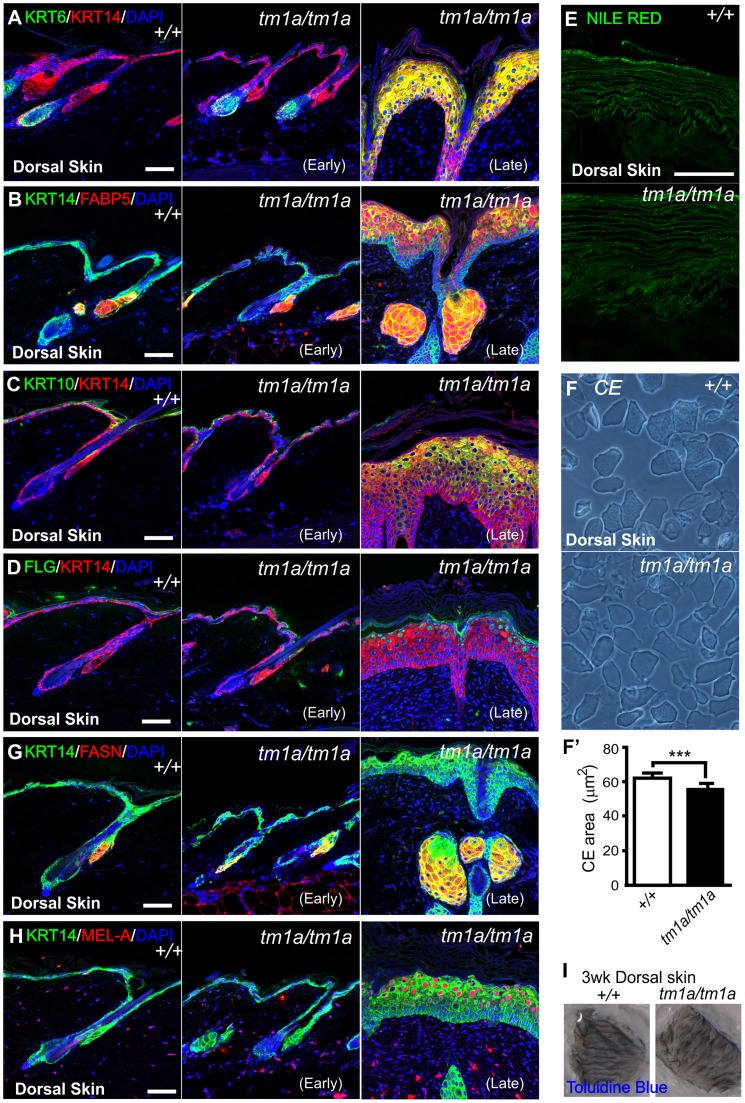
Biochemical analysis of *Krt76* disrupted skin. (A-E, G–H) Immunofluorescence (or dye) analysis of wild type, early and late phenotype *Krt76^tm1a/tm1a^* dorsal skin as indicated. (F) Isolated corneocytes from wild type and *Krt76^tm1a/tm1a^* mouse dorsal skin exhibit a modest reduction in surface area (F′) (***p = 0.0004). I) Dye exclusion assay revealed no defects in outside to inside barrier function in *Krt76^tm1a/tm1a^* mouse dorsal skin. Error bars  =  S.E.M. Scale bars represent 50 µm.

We next examined whether the skin of *Krt76^tm1a/tm1a^* mice underwent a normal program of differentiation. The basal keratin marker, Keratin 14 (KRT14) and the hair follicle expression of Keratin 6 (KRT6) [27] were normal in “early” phenotype *Krt76^tm1a/tm1a^* dorsal skin but both expanded in the interfollicular epidermis (IFE) of “late” phenotype *Krt76^tm1a/tm1a^* indicative of a wounding response (Figure 3A). Likewise, the psoriasis and wounding associated factor, Fatty acid binding protein 5 (FABP5) [28], [29], showed normal weak suprabasal IFE expression in WT and “early” phenotype *Krt76^tm1a/tm1a^* mice which increased dramatically when wounds developed in “late” phenotype *Krt76^tm1a/tm1a^* mice (Figure 3B). Keratin 10 (KRT10), a marker of the stratum spinosum, and Filaggrin (FLG), a marker of the stratum granulosum, were again normal in early phenotype *Krt76^tm1a/tm1a^* dorsal skin but expanded upon wounding in late phenotype mice (Figure 3C, D). We also surveyed lipid profiles of the cornified envelope with Nile Red, demonstrating that the deposition of extracellular lipid lamellae were unaffected in mutant animals (Figure 3E). The terminal products of epidermal differentiation, the corneocytes, also appeared to form normally, albeit with a small but significant reduction in surface area which we propose derives from hypercellularity in the epidermis (Figure 3F). While the overall differentiation of keratinocytes in late phenotype *Krt76^tm1a/tm1a^* dorsal skin was mostly normal, the hyperplasia, immune infiltrate and IFE expression of KRT6 and FABP5, were reminiscent of the hyperproliferative skin disorder, psoriasis [30], [31], [32]. We also observed enlargement of sebaceous glands shown histologically in Figure 2B and further indicated by sebocyte markers, FABP5 and FASN [33], [34] (Figure 3B, G). Hyperpigmentation was also analysed using an MELAN-A (MEL-A) antibody which revealed dermal melanocytes were abnormally increased in density in the dermis and pigment increased in late phenotype *Krt76^tm1a/tm1a^* epidermis (Figure 3H). Their location concurred with the increased incidence of pigment detected in H&E sections [35] (Fig. 2B, upper arrowhead).

The progressive deterioration of the skin in these mice led us to examine whether the barrier function and integrity of the skin was compromised as a result of loss of KRT76 function. Dorsal skin from 3 week old mice (without overt wounding) were subjected to toluidine blue dye exclusion tests and no dye penetrance was observed indicating an intact outside to inside barrier (Figure 3I). Unlike other models of intermediate filament dysfunction, we observed no evidence of cell fragility and intraepidermal cell breakages by histology. This was confirmed using tape stripping assays, which showed no increased susceptibility to dye uptake (Figure S1A) and similar yields of corneocytes in tape stripping assays (Figure S1B).

### Histological and biochemical analysis of conditional *Krt76* knockout skin

**Figure 4:**
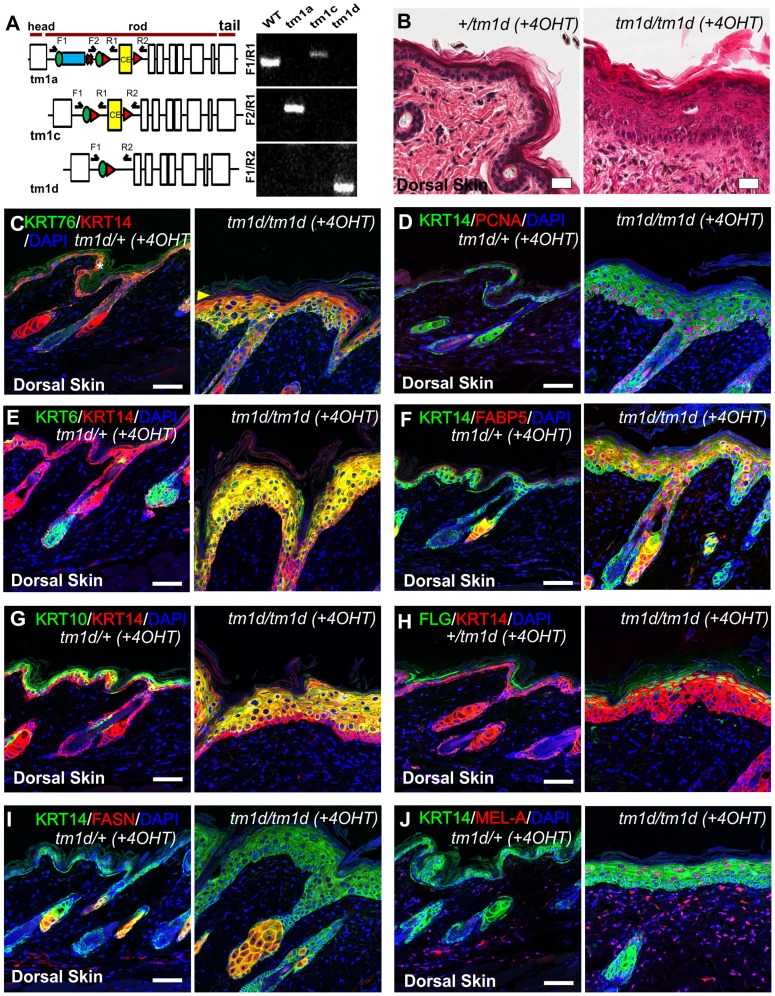
Histological and biochemical analysis of conditional *Krt76* knockout skin. (A) Exon structure and domain prediction of mouse *Krt76 gene*. Blue box represents insertion of β-galactosidase (β-gal/LacZ) cassette in the *Krt76^tm1a^* reporter allele. Green circles and red triangles indicate *frt* and *loxP* sites. Validation of the mutant alleles was achieved using PCR amplification (see Protocol S1). (B) H&E staining of dorsal skin from 4OHT-treated control and *Krt76^tm1d/tm1d^* mice. (C–J) Immunofluorescence analysis of 4OHT-treated control and *Krt76^tm1d/tm1d^* mouse dorsal skin as indicated. Yellow arrowhead indicates absence of granular layer KRT76 staining. Asterisks indicate non-specific staining. Scale bars represent 50 µm.

To further confirm that the phenotypes we observed were representative of a null allele, and to confirm the phenotype we observed was driven by gene deletion in the epidermis and not in another organ, we generated a conditional KRT76 allele by crossing these mice with a flippase expressing line to remove the LacZ and NeoR cassettes; thereby generating a *Krt76^tm1c^* allele (Figure 4A). Mice homozygous for *Krt76^tm1c^* were functionally and phenotypically wild type. This allele was then manipulated to achieve gene deletion by crossing to Cre-driver strains (Figure 4A and Protocol S1). Global gene inactivation using CMV-Cre recapitulated the gene trap phenotype, resulting in early postnatal lethality. Temporally controlled *Krt76* deletion specifically in the epidermis was achieved by topical application of 4-hydroxytamoxifen (4OHT) to the dorsal skin of 8 weeks old *Krt76^tm1c/tm1c^* mice carrying a K14-CreER transgene. These animals (*Krt76^tm1d/tm1d^*) showed regions of IFE hyperplasia and wounding after 3 weeks of treatment (Figure 4B) which was consistent with KRT76 deletion in these areas (Figure 4C, see granular layer absence indicated by arrowhead). As with *Krt76^tm1a/tm1a^* mice, hyperproliferation was increased in conditional knockouts (Figure 4D) as well as up-regulation of KRT14, KRT6 and FABP5 IFE expression (Figure 4E, F). Both KRT10 and FLG cell layers appeared to differentiate in normal sequence and showed wound related expansion (Figure 4G, H). The sebaceous glands were again enlarged, as shown by both FABP5 and FASN staining (Figure 4F, I) and like genetrap *Krt76^tm1a/tm1a^* mice, an increase in Melanin-A reactivity was seen (Figure 4J). Taken together these experiments confirm that the phenotypes we observe in these mice are due to epidermal specific knockout of KRT76.

### *Krt76* mutant mice show barrier function defects

**Figure 5:**
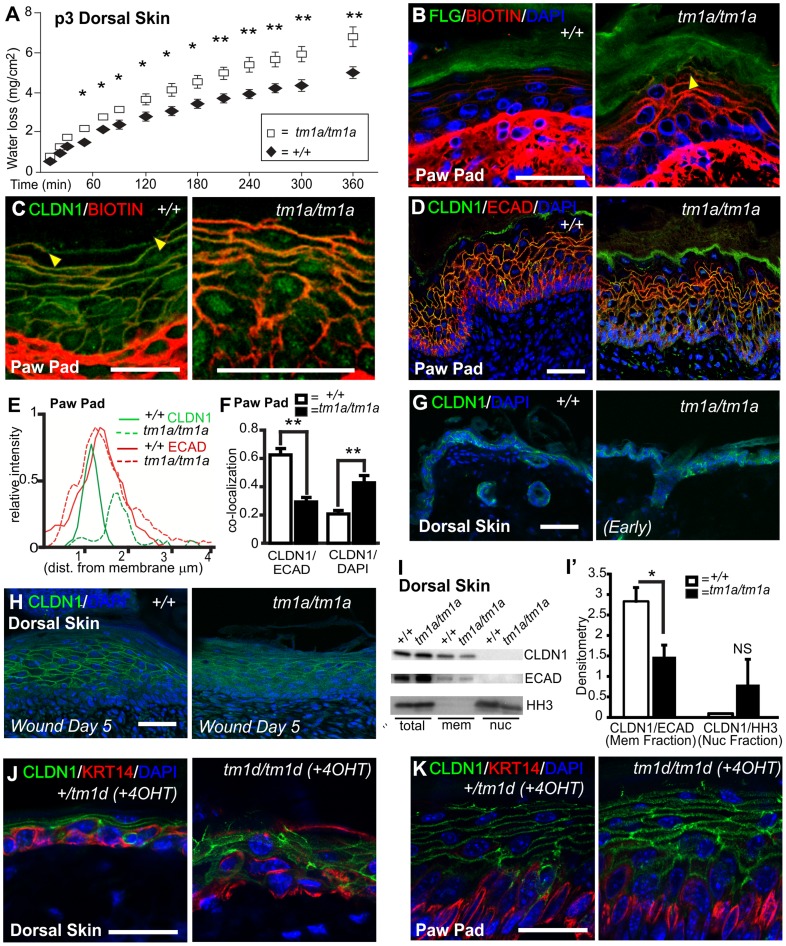
*Krt76^ mutant^* mice show barrier function defects and KRT76 stabilises Claudin1 at tight junctions. (A) Transepidermal water loss assay on P3 dorsal skin from wild type and *Krt76^tm1a/tm1a^* mice. (B) P3 paw pad skin was dermally injected with a biotin tracer and diffusion through the epidermis assessed, with Filaggrin (FLG) and DAPI co-staining for tissue orientation. Yellow arrowhead shows diffusion in suprabasal keratinocytes into cornified layer. (C) Biotin tracer was assessed alongside TJ component, Claudin1 (CLDN1). Tracer exclusion indicated by flanking yellow arrowheads. (D) Immunofluorescence analysis of CLDN1 and Ecadherin (ECAD) distribution in wild type and *Krt76^tm1a/tm1a^* mouse dorsal skin. (E) Image quantification at the cellular surface shows an inward shift and a decrease in intensity of CLDN1 not observed with ECAD. (F) Further quantification by image analysis of CLDN1 co-localisation at the cell surface with ECAD or DAPI in the nucleus. (G, H) Immunofluorescence analysis of CLDN1 localization in dorsal skin of wild-type and *Krt76^tm1a/tm1a^* mice in early phenotype and biopsy wounded adult dorsal skin of wild-type and *Krt76^tm1a/tm1a^* mice. (I) Dorsal skin fractionation assay showing localisation of different proteins to different fraction; relative lcoalisation of CLDN1 are indicated in (I′). (J, K) Immunofluorescence analysis of CLDN1 localization in adult dorsal skin and paw pads of 4OHT-treated conditional *Krt76* knock-out mice and control sibling. Note paw pad phenotype from grooming transfer of tamoxifen. *p<0.05, **p<0.01. Error bars  =  S.E.M. Scale bars represent 50 µm.

Hyper-proliferation, induction of wounding keratins, unresolved wounds, and follicular dysmorphology are phenotypes associated with a loss of barrier function. Neonatal barrier function in dorsal skin was examined using a transepidermal water loss (TEWL) assay and identified a significant defect in the cutaneous barrier in *Krt76^tm1a/tm1a^* pups compared to their control littermates (Figure 5A). Importantly, this dorsal skin defect (at P3) was apparent before obvious skin wound lesions develop. As our previous phenotypic characterisation indicated this barrier function breakdown was unlikely to be linked to overt defects in cell stability, epidermal stratification, lipid deposition or terminal differentiation we examined tight junctions (TJ). Loss of TJ functionality can result in a compromised epidermal barrier independent of defects in lipid deposition or keratinocyte differentiation [16], [17]. Furthermore, alterations in TJ proteins are an early event in psoriasis [20], a disease with phenotypes that parallel some of those evident in *Krt76^tm1a/tm1a^* and *Krt76^tm1d/tm1d^* mice. To investigate TJ integrity we subcutaneously injected P3 mouse paw pads with membrane impermeable Sulfo-NHS-Biotin and tracked its diffusion using streptavidin immunohistochemistry. In WT epidermis, the diffusion of this high molecular weight compound was restricted before the interface of the granular and cornified layers, defined by FLG expression (Figure 5B), but in *Krt76^tm1a/tm1a^* littermates the tracer was detected within the cornified layer (Figure 5B, see arrowhead). Co-staining with a cell surface marker (CLDN1) showed regions of distal dye exclusion in wild type animals (Figure 5C, see region defined by arrowheads), which were absent in mutant mice, further indicating that TJ function in these animals was disrupted (Figure 5C). The ultrastructure of TJ's in P3 paw pad was grossly normal (e.g. kissing points) and their number and position were comparable to their WT and heterozygote littermates (Figure S1C). Desmosomes also appeared normal (Figure S1C).

### *Krt76* stabilises Claudin1 at tight junctions

In assessing the diffusion of the biotin tracer in paw pad skin we noted that CLDN1 exhibited broader margins at the cell periphery and acquired a partial (albeit weak) nuclear localization (Figure 5C). This altered distribution was also observed and quantified in samples stained with CLDN1, DAPI and E-cadherin (Figure 5D, E, F), which confirmed the inward shift and partial nuclear localisation. While CLDN1 is typically a cytoplasmic protein, nuclear redistribution of CLDN1 has been previously reported [36]. Dorsal skin from young animals taken prior to the development of wounding phenotypes also exhibits mislocalisation of membranous CLDN1 (Figure 5G) and this was further exacerbated when wounds formed (Figure 5H), although CLDN1 in the nucleus was not evident at this anatomical site (Figure 5I). Mislocalisation was also confirmed in *Krt76^tm1d/tm1d^* samples (Figure 5J, K). No difference in total CLDN1 protein levels were observed in mutant skin relative to ECAD (Figure 5I) nor was there a difference in *Cldn1* mRNA expression (Figure S1D). This data collectively suggests KRT76 is required to correctly position CLDN1. Analysis of other TJ components ZO-1 and OCLN confirmed that the mislocalisation was specific to CLDN1 (Figure S2 and S3). In conclusion, our observations using several different experimental approaches indicate that KRT76 is required for normal TJ composition and in particular, the correct membrane localization of CLDN1.

### KRT76 interacts with Claudin1

**Figure 6:**
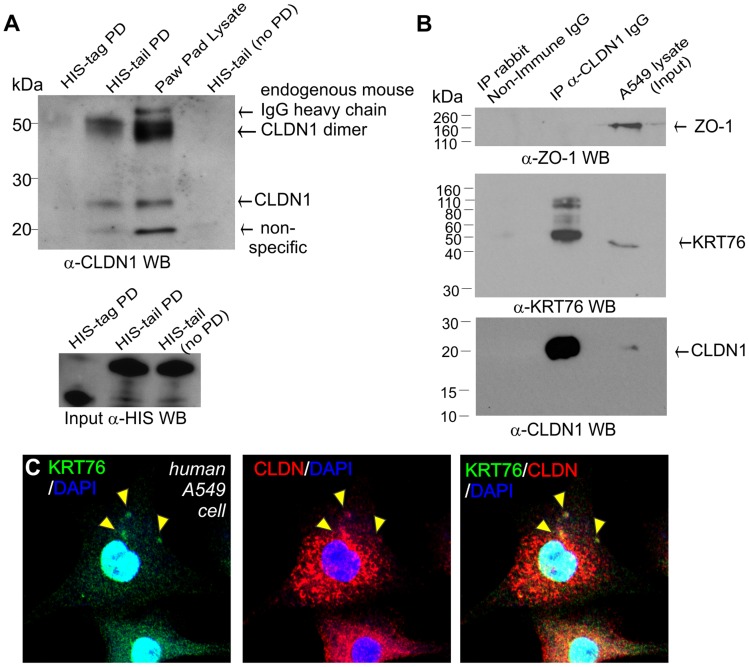
KRT76 interacts with Claudin1. (A) HIS-tagged KRT76 tail domain and HIS-tag alone where produced in E.coli, purified and immobilised on nickel-resin. Resin was then incubated with mouse paw pad lysates and the specific pull-down of CLDN1 with the KRT76- tail domain and not HIS-tag was shown by anti-Claudin1 WB. (B) Soluble extracts were prepared from A549 cells and anti-CLDN1 or non-immune IgG antibody immunoprecipitated. IP and lysate/input samples were then blotted for ZO-1, CLDN1 and KRT76. (C) A549 cells co-express CLDN1 and KRT76 and these colocalise in cytoplasmic punctate structures -see arrowheads.

Given that KRT76 is required for normal CLDN1 localization we next assessed a possible physical association between the proteins. Although KRT76 antibodies proved unsuitable for co-immunoprecipitation experiments, we were able to express the tail domain of the protein and conjugate this to nickel magnetic beads. Paw pad lysates were then applied to the beads and interacting proteins eluted. Using this approach we were able to identify a specific interaction between the tail domain of KRT76 and endogenous CLDN1 (23 kDa) and a second higher molecular weight species (∼50 kDa) which may represent previously reported CLDN1 dimers [37], [38]. No such interactions were observed with the HIS-tag control protein (Figure 6A). These bands were absent from samples containing bound HIS-tail domain protein not incubated with paw skin extracts. This assay thereby shows that KRT76 can physically complex with CLDN1 although we cannot determine if this interaction is direct or indirect. The available reagents meant that performing the reverse reaction (pull-down on CLDN1) was impossible in the mouse, so we instead employed the human A549 adenocarcinomic alveolar basal epithelial cell line which we determined to endogenously express both proteins (Figure 6B). Using these cells we were able to co-immunoprecipitate KRT76 with CLDN1. Furthermore, ZO-1 (another TJ component) did not form part of this interaction complex, indicating the interaction between KRT76 and CLDN1 is specific amongst TJ components (Figure 6B). CLDN1 and KRT76 were also observed to co-localise in punctate structures within the cytoplasm of A549 cells (Figure 6C, see arrowheads). These interaction and co-localization assays confirm a physical complex between CLDN1 and KRT76 which we propose is important for mediating the barrier dysfunction and wound healing phenotypes of the KRT76 knockout mouse.

## Discussion

The keratins are classically regarded as structural proteins whose role is to form the fabric of the cytoskeleton and to stabilise epithelial cells. However, this somewhat simplistic view has increasingly been challenged by the description of their specialised and dynamic functions in a number of cellular and developmental contexts. The keratins are the most diverse class of intermediate filament proteins and in many cases their functions are poorly defined. In this study we describe the characterisation of KRT76, one of the least understood of the protein family, delineating its essential role in the maintenance of the integrity of the skin. Under resting conditions, *Krt76* is expressed at its highest levels in the paw, oral epithelium and vagina, localising to the granular layer. It is also expressed in the dorsal epithelium, particularly during the late stages of embryonic development. Wounding induces *Krt76* expression, although the profile of this induction is distinct from other wounding keratins like *Krt6* and *Krt16*.

To examine the functional relevance of this expression and its role in epidermal homeostasis we inactivated the gene in mice globally and in a skin specific manner. Loss of *Krt76* results in the rapid appearance of extensive non-healing wounds (especially at sites of active grooming), and the subsequent infection of these lesions contributes significantly to morbidity and mortality in the mice. Unlike other knockout models of structural keratins we failed to observe cytolysis and/or blistering in the skin. Instead we observed a relatively unperturbed program of keratinocyte differentiation although this gives way to a phenotype of hyperproliferation as the phenotype of the animals worsens. What triggers this change remains to be determined, however the frequency of wounds around active grooming sites suggests that KRT76 deletion may impair the ability of the skin to recover from physical insults normally experienced in the life of the mouse. This theory is supported by the demonstration that induced wounds in the skin of these mice, administered prior to the accumulation of significant cutaneous damage, failed to heal normally.

As well as the progressive wounding phenotype observed in these mice, we also noted cellular changes which were consistent with defects in the barrier function of the epidermis. This was confirmed using trans-epidermal water loss assays in neonatal animals. We were unable to establish a role for defective keratinocyte stability or termination in driving this defect, nor was lipid transport affected in the mice to any appreciable level. Instead, we observed the specific mislocalisation of the TJ component CLDN1, even in newborn mice and in animals without overt or severe cutaneous defects. Indeed previous reports have shown that even significant hyperproliferation induced by two step carcinogenesis treatments is unable to elicit similar changes [39]. Although TJs appeared normal at an ultra-structural level, their reduced capacity to limit the movement of molecules between differentiating keratinocytes in our mice suggests that they were functioning abnormally. Importantly mislocalisation was not observed for other structural elements of the TJ. It is therefore notable that the phenotype of the *Krt76* KO mice is strikingly similar to animals carrying homozygous mutations in *Cldn1* [17]. In both cases, barrier function defects are detectable by biotin tracer and TEWL assays (but not by dye exclusion), and both have apparently normal formation of TJ structures as assessed by EM. Overall, the phenotypes of *Krt76* null mice are somewhat milder than their CLDN1 counterparts, suggesting that despite loss of KRT76, some CLDN1 can still contribute to partial TJ function. By studying both skin extracts and cell lines endogenously expressing both CLDN1 and KRT76 we were able to demonstrate a physical association between these proteins, mediated by the tail domain of the latter. At present we do not know whether this interaction is direct, or whether the proteins exist in a larger complex. In either case, the loss of KRT76 is clearly required for normal tight junction function and for CLDN1 localisation. Although links between the tight junction and the cytoskeleton have been described for actin, this is the first report detailing an interaction with the keratin intermediate filaments.

In summary, we believe that the KRT76 protein represents a new and essential protein required for maintaining epidermal integrity. Its expression during fetal development and during wound healing suggests it is required to establish and/or stabilise the development of TJs in differentiating keratinocytes, specifically through mediating the correct localisation off CLDN1 to these structures. Deletion of the protein leads to defects in TJ function that are at least in part associated with the development of progressively worsening wounds. Whether this severe later phenotype, which ultimately leads to the death of the animals, reflects a separate, non-TJ, role for the protein in wound repair is unclear. Mislocalization of CLDN1 is a feature of a number of cutaneous diseases such as psoriasis [40], and in a number of cancers [36], [41], [42]. KRT76 depletion has also been linked with human oral carcinomas and premaligant epidermal changes [43]. It will therefore be interesting to determine the extent to which this new cytoskeletal-TJ interface between CLDN1 and KRT76 interaction plays a role in the development or progression of these diseases.

## Materials and Methods

### Ethics statement

Animal models were maintained under the auspices of ethics applications to Monash University and subject to the conditions of the Australian Bureau of Animal Welfare.

### Transgenic mice

*Krt76^tm1a/tm1a^* mice were generated in the Mouse Genetics Programme at the Wellcome Trust Sanger Institute [24]. Animals were bred and maintained on a mixed background of C57BL/6J^TyrcBrd^; C57BL/6N. The *Krt76^tm1a/tm1a^* characterisation data presented is available at www.mousephenotype.org. Targeting vector information is available at http://www.mousephenotype.org/martsearch_ikmc_project/martsearch/ikmc_project/38047. Flip recombinase (Flipper) mice [44], K14-CreER mice [45] and CMV-Cre mice [46] have been described previously. 1.5 mg of 4-hydrotamoxifen (H6278, Sigma-Aldrich) was applied to a shaved region of lower back skin in 100 µl of acetone every second day for 21 days before mice were harvested for analysis.

### Genotyping transgenic mice

The PCR conditions were set for amplification of small PCR fragments only. Details of primer sequences, reaction composition and cycling profile are provided in Protocol S1.

### Histological preparation and staining

Staining for LacZ expression was performed as previously described [47] on frozen sections and counterstained with Nile Red. Immunofluorescence experiments were performed after citrate based antigen retrieval. Primary antibodies were ZO-1 (Invitrogen cat# 339100), Occludin (BD Transduction cat# 611090), Claudin-1 (ABCAM cat# ab15098), cytokeratin14 - LLOO2 (ABCAM cat # ab7800), keratin10 (Covance PRB-159P), keratin6 (Covance cat # PRB-169P), Ecadherin (Life Technologies, 13-1900), phospho-histone H3 (Cell Signalling, #9708), PCNA (Santa Cruz Biotechnology sc-9857), CLDN1 (Santa Cruz Biotechnology, sc-81796), Keratin 76 (Sigma-Aldrich HPA019696) and Keratin 76 (Sigma-Aldrich HPA019656), Filaggrin (FLG- Covance PRB-417P), FASN (Santa Cruz Biotechnology, sc-48357), and Melan-A (MEL-A, Santa Cruz Biotechnology, sc-20032). All secondary antibodies were AlexaFluor conjugated (Invitrogen).

### Imaging and analysis

Sections were imaged using Lecia SP5 5 Channel, Olympus FV500 confocal microscopes or Aperio slide scanners. Bright field images of wound healing experiments were taken with Olympus dotslide brightfield microscope. Images for CLE assays were acquired with Olympus CKX41 and exported to FIJI software [48] for cell analysis.

### Wound healing experiments

Mice (age-matched males; 6 weeks) were isofluorane-anaesthetized and 2 full-thickness excisional wounds were made with a 5 mm biopsy punch (Livingstone International). Wound tissue was harvested with an 8 mm biopsy punch.

### qRT-PCR

One µg of DNase (Ambion) treated RNA was used for cDNA synthesis (SuperScriptVILO). Multiplex quantitative PCR was performed using Taqman probes for *Gapdh* (VIC-primer limited labelled, cat# 4448484) and *Krt76* (FAM labelled, Cat#4351372) with TaqMan Fast Advanced Master Mix Protocol (PN 4444605B). Gene specific primers were designed and used in conjunction with SYBR Green PCR Master Mix (Applied Biosystems) for the detection and quantification of *Claudin1* (5′-ATTTCAGGTCTGGCGACATT-3′ fwd, 5′-ACACCTCCCAGAAGGCAGAG-3- rev) , *Krt6b (5* ′*-*CAGACCCCAGATACCCTGGC-3′ fwd, 5′-GAGCAGAGATGGCATCATGTGAGCAACAGG-3′ rev) , *Krt16* (5′- AACAGCCTAGAAGAGACCAAAGGC-3′ fwd, 5′-GGTAGGGGAGACAGATGGGGAATGCGC-3′ rev) mRNA as compared to *Gapdh* (5′- CTGCACCACCAACTGCTTAG-3′ fwd,5′- GTCTTCTGGGTGGCAGTGAT-3′ rev).

### Protein fractionation

All fractionation experiments were performed on dorsal epidermis of P3 animals. Pups were euthanized (Pentobarbital) and skin was removed as previously described [49]. Skins were floated on 2.3 U/mL Dispase (Life Technologies) in PBS overnight at 4°C. The epidermis was separated from the dermis and protein fractionated using a Qproteome Cell Compartment Kit (Qiagen). Western blots for E-cadherin (Life Technologies) and total Histone H3 (Cell Signaling) were performed on the nuclear and membrane fractions. Image Quant software was used to calculate densitometry and quantify protein levels. Claudin-1 levels in the membrane fraction were normalized to E-cadherin for each sample.

### Biotin tracer assays

TJ permeability assays were undertaken as previously described [17], [50]. Briefly, a solution of 10 mg/ml EZ-Link Sulfo-NHS-LC-Biotin (Pierce) in PBS containing 1 mM CaCl_2_ was injected into the paw pads of P3 pups. Paw pads were incubated at room temperature for 30 minutes prior to frozen sectioning and IHC with conjugated Streptavidin Alexafluor 594 (Life Technologies, S-11227).

### Ultrastructural analysis

Tissue was fixed in Karnovsky's fixative (2% paraformaldehyde, 2.5% glutaraldehyde in 0.1 M Cacodylate buffer) for 2 hours. Then washed in 3×10 min changes of 0.1 M Cacodylate buffer. Post-fixation was with 2% osmium tetroxide in 0.1 M Cacodylate buffer followed by dehydration through a graded series of alcohols, two acetone rinses and embedding in Spurrs resin. 80 nm sections were cut with a diamond knife (Diatome, Switzerland) on an Ultracut-S ultramicrotome (Leica, Mannheim, Germany) and contrasted with uranyl acetate and lead citrate. Images were captured with a Megaview II cooled CCD camera (Soft Imaging Solutions, Olympus, Australia) in a JEOL 1011 transmission electron microscope.

### HIS-tag Pull-down from Mammalian Lysates

Recombinant HIS-tagged proteins were produced by IPTG induction (0.4 mM) of T7 Express lysY/I^q^ Competent *E.coli* (New England Biolabs C3013I) transformed with HIS-tag expressing control vector, pET-30a+ (Novagen) or HIS-tagged KRT76 domains in pDEST17 gateway backbone (Life Technologies), grown for 6–8 hours at 37°C in low salt LB, supplemented with 100 µg/ml ampicillin or 50 µg/mL kanamycin (as required). Recombinant protein was purified using 0.1 ml per 1 ml of culture of PopCulture lysis reagent (Novagen), 1 µl per mL of culture of 40 U/ml of Lysonase bioprocessing reagent (Novagen), protease inhibitors (Sigma P8849), and His-Mag beads (Novagen) according to manufacturer's protocols. Bound recombinant HIS and HIS-KRT76 protein were washed and stored at 4°C as a 1∶2 resin slurry in Tris-saline pH 7.4 containing protease inhibitors. Paw pad skin of adult was collected in RIPA lysis buffer and incubated with HIS or HIS-KRT76 overnight at 4°C. HisMag bead-bound HIS and HIS-KRT76 + lysates were then washed four times in Tris-saline pH 7.4 including 1% Triton X-100 and immunoblotted for mCLDN1 (Santa Cruz Biotechnology, SC-81796) and the HIS tag (Sigma-Aldrich, clone HIS-1).

### A549 analysis and CO-IP

A549 cells (ATCC CCL-185) were cultured in low-glucose DMEM including 10% FCS, Penicillin Streptomycin and L-glutamine. For CO-IP, cells at confluence were scraped and lysed in 1% Triton X-100 in 1xTBS with Roche complete protease inhibitor tablet, extracted for 2 hrs at 4°C then supernatant collected. The supernatant was applied to binding columns prepared using the Pierce Crosslink IP Kit and CO-IP performed as per manufacturers protocol. Bound fractions were washed 3 times in lysis buffer before elution and standard WB analysis. For Immunofluorescence, 2×10^5^ cells were seeded on Collagen type 1 coated glass coverslips in 6 well plate format and processed as previously described [51].

### Dye exclusion

E18.5 embryos or 3 week old dorsal skin were collected and transferred through a Methanol gradient with emersion for 1 minute each: 25% methanol in water, 50% methanol in water, 75% methanol in water, 100% methanol, 75% methanol in water, 50% methanol in water, 25%methanol in water and equilibrated in PBS. All reagents were chilled. Tissue was then exposed to 0.1% Toluidine Blue solution in water for 2 minutes and destained in 1xPBS pH7.4. For tape stripping, clipped dorsal skins were first tape stripped twelve times with adhesive tape before tissue collection.

### Cornified envelope assay

Analysis of the size of corneocytes in the cornified lipid envelope (CLE) assay was performed as previously published [52].

### Statistical analysis

Statistical analysis was performed using unpaired students test, values of p<0.05 were deemed significant. A minimum of 3 mice were analysed per condition unless otherwise stated. In graphs, error bars represent Standard Error of the Mean (S.E.M).

## Supporting Information

Figure S1 Tape stripping and TJ ultrastructure. (A) Dye exclusion assay with and without tape stripping revealed no fragility in *Krt76^tm1a/tm1a^* mouse dorsal skin. (B) Quantification of corneocyte yield in tape stripping assay (Figure 3F). (C) Electron micrographs of TJ ultrastructure in wild type and *Krt76^tm1a/tm1a^* mice. TJ =  tight junctions, D =  desmosome. (D) *Cldn1* mRNA qRT-PCR analysis of p3 paw pad skin relative to *Gapdh* (n = 3), ns  =  not significant. Error bars represent S.E.M.
(TIF)

Figure S2 ZO-1 at tight junctions. (A) Immunofluorescence analysis of ZO-1 demonstrates co-expression with KRT76 in suprabasal cells of the paw pad. (B, D) No obvious changes were detected in ZO-1 localization in paw pads of *Krt76^tm1a/tm1a^* mice. (C, D) Immunofluorescence analysis of ZO-1 in wild type and wounded *Krt76^tm1a/tm1a^* mouse dorsal skin showed only a modest change in intensity. (E, F) In contrast to CLDN1, ZO-1 changes in dorsal skin correlate with wounding and not *Krt76* disruption. Scale bars represent 50 µm.
(TIF)

Figure S3 Occludin at tight junctions. (A, B) Immunofluorescence analysis of Occludin (OCLN) demonstrates an increase in intensity are cell margins in the paw pads of *Krt76^tm1a/tm1a^* mice. (C) Immunofluorescence analysis of OCLN in conditional *Krt76* mouse dorsal skin shows OCLN is not expressed in dorsal skin nor wounded dorsal skin, but is detected in the hair follicle. Scale bars represent 50 µm.
(TIF)

Protocol S1 This file details the primer sequences and amplification conditions employed to genotype the different Krt76 alleles employed during this study.
(XLSX)
