# Supplementary figures and images for: Keratin 76 Is Required for Tight Junction Function and Maintenance of the Skin Barrier
Source: PLoS Genet. 2014 Oct 23;10(10):e1004706. doi: 10.1371/journal.pgen.1004706 (PMC4207637; doi:10.1371/journal.pgen.1004706)

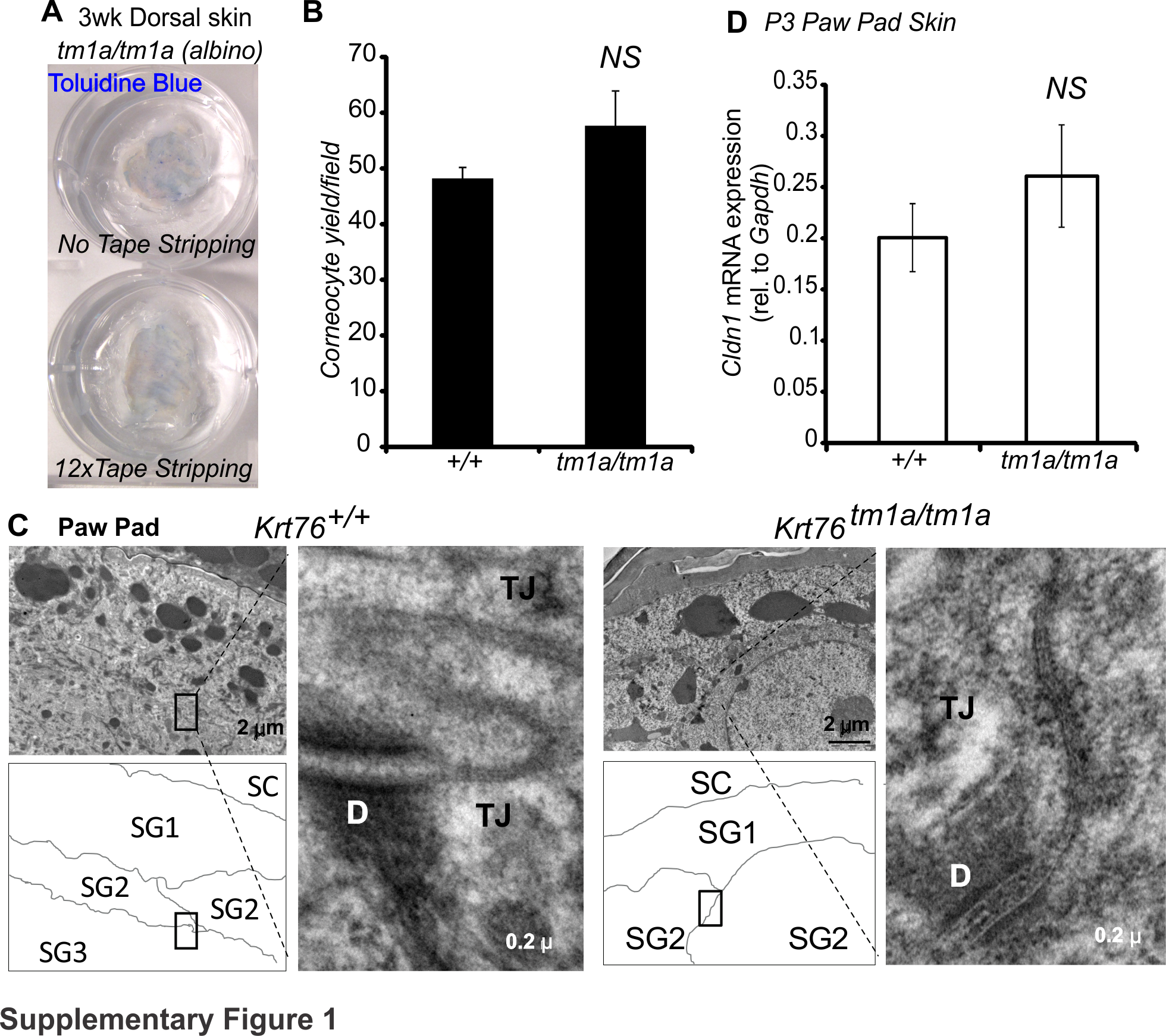

Supplement: Figure S1 — Tape stripping and TJ ultrastructure. (A) Dye exclusion assay with and without tape stripping revealed no fragility in Krt76tm1a/tm1a mouse dorsal skin. (B) Quantification of corneocyte yield in tape stripping assay (Figure 3F). (C) Electron micrographs of TJ ultrastructure in wild type and Krt76tm1a/tm1a mice. TJ = tight junctions, D = desmosome. (D) Cldn1 mRNA qRT-PCR analysis of p3 paw pad skin relative to Gapdh (n = 3), ns = not significant. Error bars represent S.E.M. (TIF) [file pgen.1004706.s001.tif]

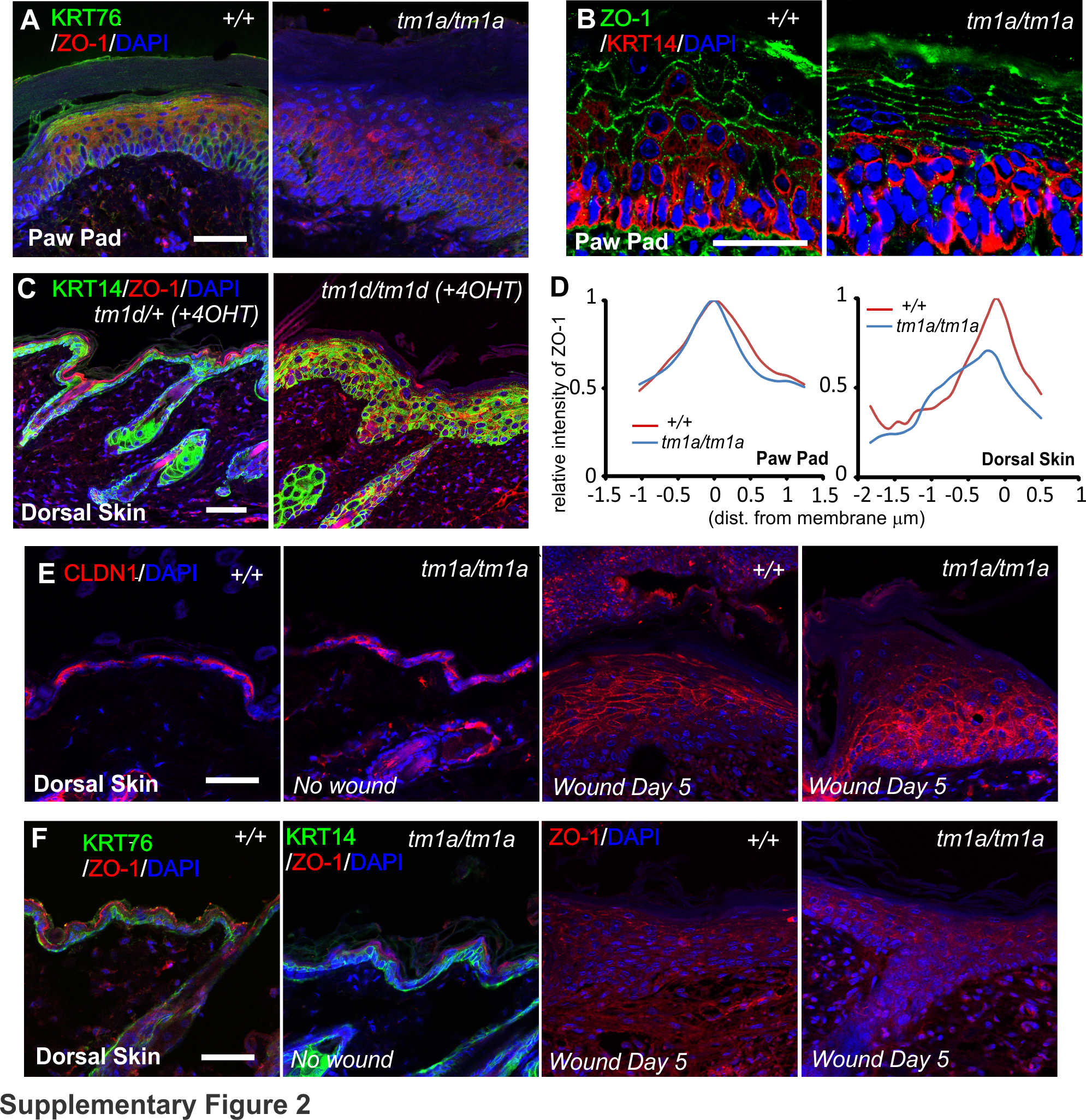

Supplement: Figure S2 — ZO-1 at tight junctions. (A) Immunofluorescence analysis of ZO-1 demonstrates co-expression with KRT76 in suprabasal cells of the paw pad. (B, D) No obvious changes were detected in ZO-1 localization in paw pads of Krt76tm1a/tm1a mice. (C, D) Immunofluorescence analysis of ZO-1 in wild type and wounded Krt76tm1a/tm1a mouse dorsal skin showed only a modest change in intensity. (E, F) In contrast to CLDN1, ZO-1 changes in dorsal skin correlate with wounding and not Krt76 disruption. Scale bars represent 50 µm. (TIF) [file pgen.1004706.s002.tif]

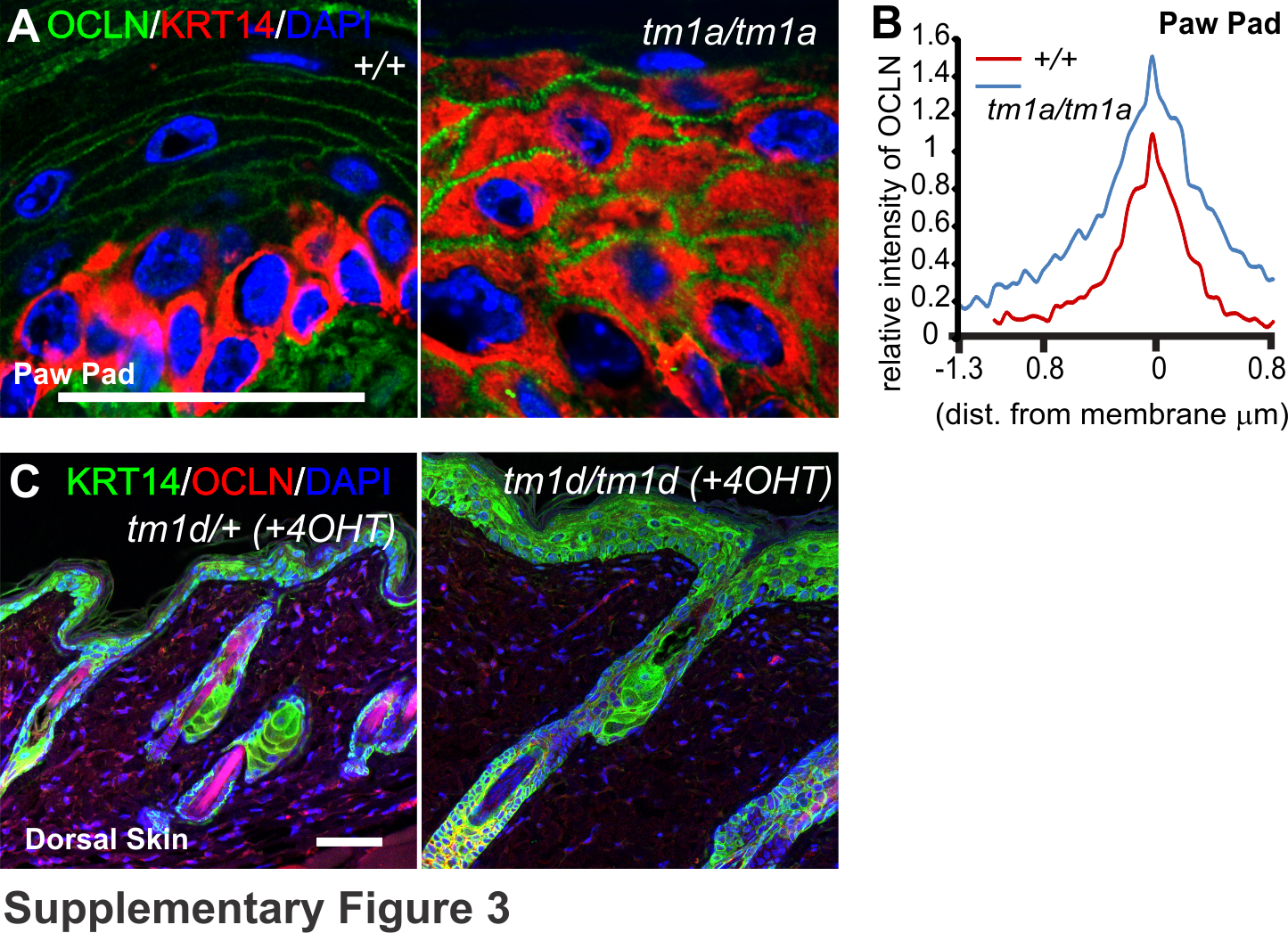

Supplement: Figure S3 — Occludin at tight junctions. (A, B) Immunofluorescence analysis of Occludin (OCLN) demonstrates an increase in intensity are cell margins in the paw pads of Krt76tm1a/tm1a mice. (C) Immunofluorescence analysis of OCLN in conditional Krt76 mouse dorsal skin shows OCLN is not expressed in dorsal skin nor wounded dorsal skin, but is detected in the hair follicle. Scale bars represent 50 µm. (TIF) [file pgen.1004706.s003.tif]
